# Assessing Potential Habitat and Carrying Capacity for Reintroduction of Plains Bison (*Bison bison bison*) in Banff National Park

**DOI:** 10.1371/journal.pone.0150065

**Published:** 2016-02-24

**Authors:** Robin Steenweg, Mark Hebblewhite, David Gummer, Brian Low, Bill Hunt

**Affiliations:** 1 Wildlife Biology Program, College of Forestry and Conservation, University of Montana, Missoula, Montana, United States of America; 2 Resource Conservation, Banff National Park, Parks Canada, Banff, Alberta, Canada; Centre for Cellular and Molecular Biology, INDIA

## Abstract

Interest in bison (*Bison bison*, *B*. *bonasus*) conservation and restoration continues to grow globally. In Canada, plains bison (*B*. *b*. *bison*) are threatened, occupying less than 0.5% of their former range. The largest threat to their recovery is the lack of habitat in which they are considered compatible with current land uses. Fences and direct management make range expansion by most bison impossible. Reintroduction of bison into previously occupied areas that remain suitable, therefore, is critical for bison recovery in North America. Banff National Park is recognized as historical range of plains bison and has been identified as a potential site for reintroduction of a wild population. To evaluate habitat quality and assess if there is sufficient habitat for a breeding population, we developed a Habitat Suitability Index (HSI) model for the proposed reintroduction and surrounding areas in Banff National Park (Banff). We then synthesize previous studies on habitat relationships, forage availability, bison energetics and snowfall scenarios to estimate nutritional carrying capacity. Considering constraints on nutritional carrying capacity, the most realistic scenario that we evaluated resulted in an estimated maximum bison density of 0.48 bison/km^2^. This corresponds to sufficient habitat to support at least 600 to 1000 plains bison, which could be one of the largest 10 plains bison populations in North America. Within Banff, there is spatial variation in predicted bison habitat suitability and population size that suggests one potential reintroduction site as the most likely to be successful from a habitat perspective. The successful reintroduction of bison into Banff would represent a significant global step towards conserving this iconic species, and our approach provides a useful template for evaluating potential habitat for other endangered species reintroductions into their former range.

## Introduction

Few free-ranging wild bison (*Bison bison*, *Bison bonasus*) populations currently occur in North America (n = 27) and Eurasia (n = 36)[1,2], and there has been growing interest in restoring the species to other portions of historic range [3–6]. It has been mainly through reintroductions that conservation efforts have brought bison back from near extinction on both continents [1,7], but in North America, they occupy less than 1% of their historical range [6]. Bison were extirpated from the wild across most of the species’ former range, although there are now an estimated half-a-million bison present in North America, mostly in captive herds [8]. In Canada, wild plains bison (*Bison bison bison*) were completely extirpated in the late 19th century [8] and they are designated as “threatened” by the Committee On the Status of Endangered Wildlife In Canada (COSEWIC) [9]. Despite the 2005 recommendations of COSEWIC, plains bison are not yet listed on Schedule 1 of the Species At Risk Act (SARA), primarily because of potential economic implications for the Canadian agricultural bison industry [10]. Plains bison, therefore, are not currently protected under SARA and a national recovery strategy is not required, legally; however, the majority of wild, plains bison in Canada occur within National Parks where they are protected under the Canada National Parks Act [11].

Originally ranging across the Great Plains and into the Rocky Mountains [12], plains bison now occupy less than 0.5% of their former range in North America [1]. Currently, there are only 5 wild subpopulations in Canada, the largest of which, the Pink Mountain population, resides outside their historical range. All 5 of these subpopulations are < 1000 bison, emphasizing the importance of establishing new large populations for conservation [13]. For example, amongst the two largest bison populations in Canada are Elk Island National Park, Alberta, with ~500 bison and Pink Mountain, British Columbia with ~1000 plains bison [9]. The largest threat to the recovery of plains bison is the lack of habitat due to broad-scale conversion of suitable areas from grassland to agriculture and urban areas, land uses that are considered incompatible with bison [9]. Although there is no legal requirement for a recovery strategy, conservation actions are being planned to contribute to recovery of plains bison. Established populations are unlikely to be able to expand or successfully disperse because contiguity of suitable habitat is limited and surrounding areas are often managed to exclude bison. Thus, reintroduction of bison into previously occupied areas of suitable habitat is necessary to aid the long-term conservation and recovery of plains bison in Canada.

There is archaeological and historical evidence of bison inhabiting Banff National Park (Banff) [14] and the species likely exerted important roles in the ecosystems along the eastern slopes of the Rocky Mountains. Under the Canada National Parks Act, Parks Canada is required to maintain and, where feasible, restore the natural condition of each park, which includes the composition and abundance of native species [11]. The present Banff management plan provides direction to reintroduce a breeding population of the extirpated plains bison after the concerns of stakeholders and neighboring jurisdictions have been addressed [15]. Reintroducing plains bison in Banff could contribute to the global conservation of the subspecies by expanding its range, contributing an additional subpopulation of potentially large size, and adding to the overall population size. Here, we develop a habitat suitability index model and model of potential population size to evaluate the feasibility of bison reintroduction to accomplish these goals.

Feasibility and risk need to be assessed prior to proceeding with any reintroduction [16]. One important step towards assessing the feasibility of a reintroduction is evaluating if there is sufficient habitat for a breeding population in the proposed reintroduction area. This is particularly challenging for species like bison that have been extirpated from a potential reintroduction site for so long that no information exists on their former habitat relationships. Despite archaeological evidence for bison in Banff, it is unknown whether this area was inhabited year round by bison or may have only been used on a seasonal basis. Moreover, Banff was near the western distributional edge for bison; bison did not occur west of the continental divide in Canada [4]. Whether Banff harbored year-round resident bison populations is unclear from the archaeological evidence [14]. Thus, despite some support for reintroducing bison, it is first important to test whether Banff contains sufficient bison habitat, in both winter and summer seasons, to sustain a significantly large bison population to be both viable, and, contribute to improving their conservation status [6]. Defining sufficient habitat to sustain a significant enough bison population size is itself challenging. Here, we adopt IUCN recommendations developed from previous studies that suggested that wild free-ranging bison populations > 400 would constitute a large contribution to global conservation of bison, and that > 1000 would constitute an exceptional contribution [6,13]. Population sizes > 400 would also prevent the most serious concerns about maintaining genetic variation [17], and maintain population viability [13]. Quantifying habitat availability in both seasons is important to test whether Banff could support a year-round bison population, or, alternatively, whether seasonal habitat limitations may promote migration outside of Banff. However, as noted above, it is difficult to assess habitat suitability for a long-extirpated species [18]. In these cases, expert-based habitat suitability models are often used to summarize previous scientific literature and make predictions about potential habitat [19]. Previous studies preceding bison reintroduction have used a combination of expert-based habitat suitability indices or landcover assessments to identify potential bison habitat for restoration [5].

Plains bison are habitat generalists that can persist in many different grassland and forest types [6]. They are primarily grazers and select habitat with good graminoid forage [20], low snow depth [21] and recent burn history [22]. Our first question was to identify potential habitat for the eastern slopes region of Banff and to predict the suitability and spatial distribution of habitat in winter and summer. Habitat can be a confusing term in ecology because of the use of two diverging definitions. Habitat is often considered synonymous with static representations of specific resource, such as vegetation types following the forestry-based habitat type concept [23]. This definition is conceptually challenging for species that include non-vegetation components as conditions necessary for survival and persistence [24]. Here we adopt, therefore, the second common use of habitat, where habitat is defined as the spatial representation of the species’ niche in geographic space, where habitat is the area in which resources and conditions permit survival and reproduction of a species [24–26]. Thus in the second definition, habitat suitability is analogous with habitat quality.

Following the identification of the distribution of suitable habitat, it is important to consider the potential population size for a reintroduced population. This helps managers evaluate the potential for a reintroduced population to persist in the long-term and contribute to species recovery goals, and to plan for appropriate scale and complexity of future management actions. In the case of plains bison, most current populations are small, < 400, and understanding if there was potential habitat to support bison populations of > 400, or > 1000 would contribute significantly to their global conservation status according to the IUCN [6,13]. Thus, our second question was to estimate potential population size of bison (*i*.*e*. carry capacity) using a nutritional approach in Banff. We estimated ecological carrying capacity, K, defined as the nutritional-based number of animals that can be sustained with zero population growth, *i*.*e*. when birth and death rates are equivalent. This definition is often confused with economic carrying capacity, which under the assumption of linear density-dependence in population growth rate, is usually 50% of K, and represents the population size at which population recruitment (productivity) is maximized [26,27]. Ecological carrying capacity (K) is rarely experienced by large herbivores because of other ecological constraints such as predation and winter severity. Although K represents an idealized and likely unrealistic maximum population estimate, it is a critical parameter to understand for its over-arching limits on future population growth.

There have been numerous approaches to estimate potential population carrying capacity for ungulates [28–30]. Early and simplistic approaches project ungulate population size based on domestic cattle models with simple energetic requirements and no consideration of competition with native ungulates, climate, predation or social tolerance [31]. This approach often acts as a starting point to which additional constraints can be added, such as forage quality [28] or seasonal variation in forage quality [32]. More complicated models incorporate costs of movement, foraging, resting, traveling through snow, foraging in snow, and landscape ecology constraints such as minimum patch sizes, distances between patches, etc. [33–35]. The most recent advances combine Resource Selection Function models (RSF) as constraints on nutritional availability [36]. Despite the recent emphasis of the role of summer vegetation in determining ungulate population dynamics, little is known about summer forage for the nutritional ecology and population dynamics of bison. We focused, therefore on winter, assuming that this was the season that sets the upper limit on bison population size [28,29].

Our two main objectives were to identify bison habitat and to estimate potential population size in the proposed reintroduction area in Banff. We evaluated whether there would be sufficient year-round habitat for the bison population to exceed the IUCN guidelines of populations > 400 contributing the most to conservation [13]. It is possible that habitat could be inadequate in winter because of snow-depth effects on forage availability, in which case the reintroduction area in Banff would not be able to support a large, year-round bison population. Answering these questions is important for management because if there is insufficient amount and quality of habitat during winter in Banff, bison may migrate outside Banff more frequently in search of suitable habitat [37]. To evaluate bison habitat, we first developed a Habitat Suitability Index (HSI) model [38] based on a review of the scientific literature, an existing forage quality model, and 3 winter severity scenarios. We then validated the performance of this model with archaeological sites with bison remains. Second, using the bison winter HSI model, existing datasets on spatial ungulate forage biomass, and literature values of bison diet composition and energetic requirements, we estimated the potential winter population size of bison under different grazing intensity scenarios. Our approach provides a valuable example of evaluating potential habitat for large herbivore reintroductions to areas of their former range.

## Material and Methods

### Study area

Banff National Park (Banff) is located in the Canadian Rocky Mountains and is characterized by extreme mountainous terrain (elevation: 1400–3600 m) with large valleys that are 2–5 km wide. Winters are long and cold while summers are short and dry [39]. Banff contains all large carnivores that were present before European settlement: wolves (*Canis lupus*), grizzly bears (*Ursus arctos*), black bears (*Ursus americanus*), and cougars (*Puma concolor*). Ungulates present include elk (*Cervus elaphus canadensis*), white-tailed deer (*Odocoileus virginianus*), mule deer (*Odocoileus hemionus*), moose (*Alces americanus*), bighorn sheep (*Ovis canadensis*), and mountain goats (*Oreamnos americanus*). Two ungulates no longer present in Banff are bison and the recently extirpated caribou (*Rangifer tarandus*; [40]).

**Fig 1:**
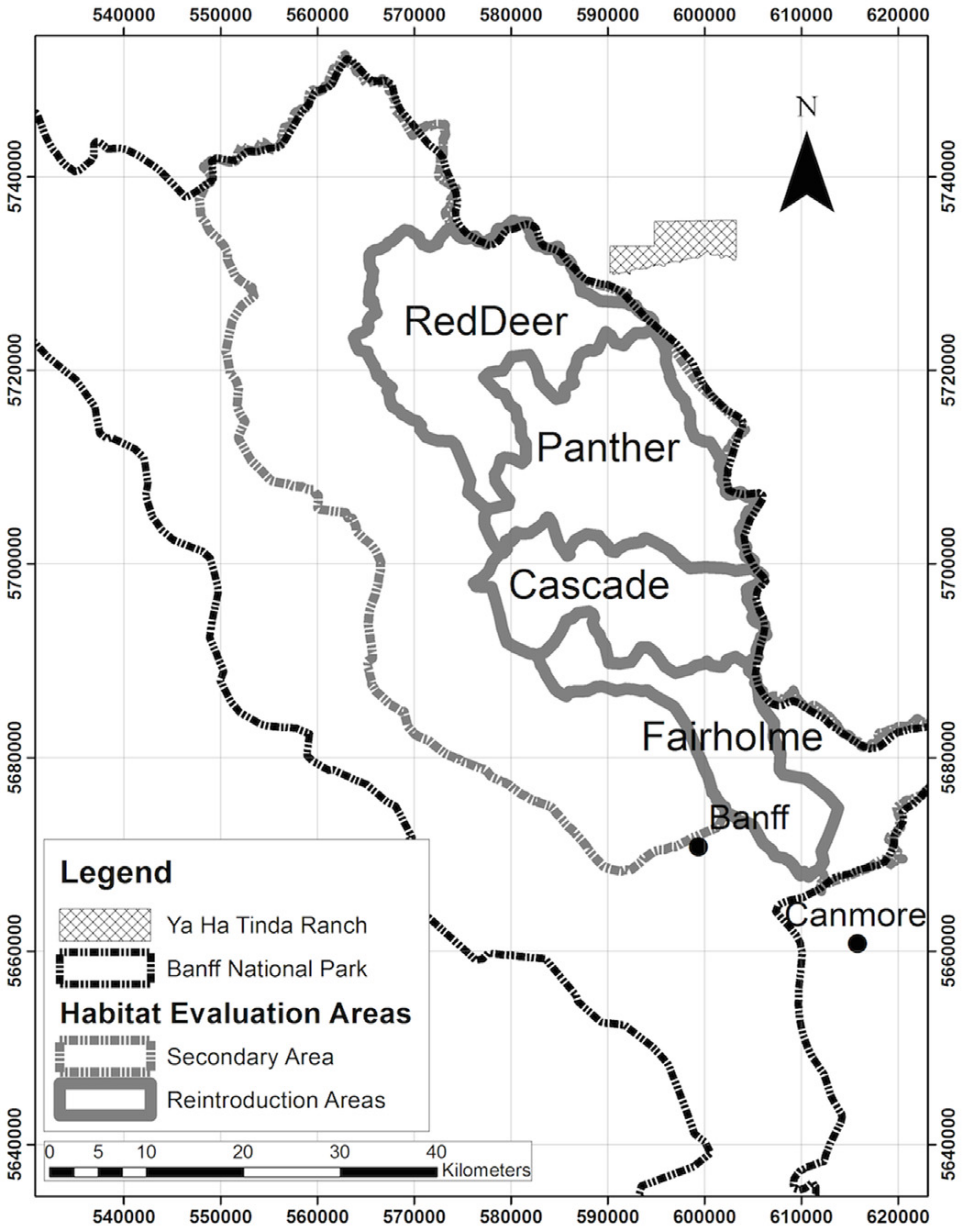
Eastern Banff National Park area that is being considered for plains bison (*Bison bison bison*) reintroduction. Within the primary reintroduction area, we evaluated 4 secondary areas: Red deer, Panther-Dormer, Cascade and Fairholme.

**Fig 2:**
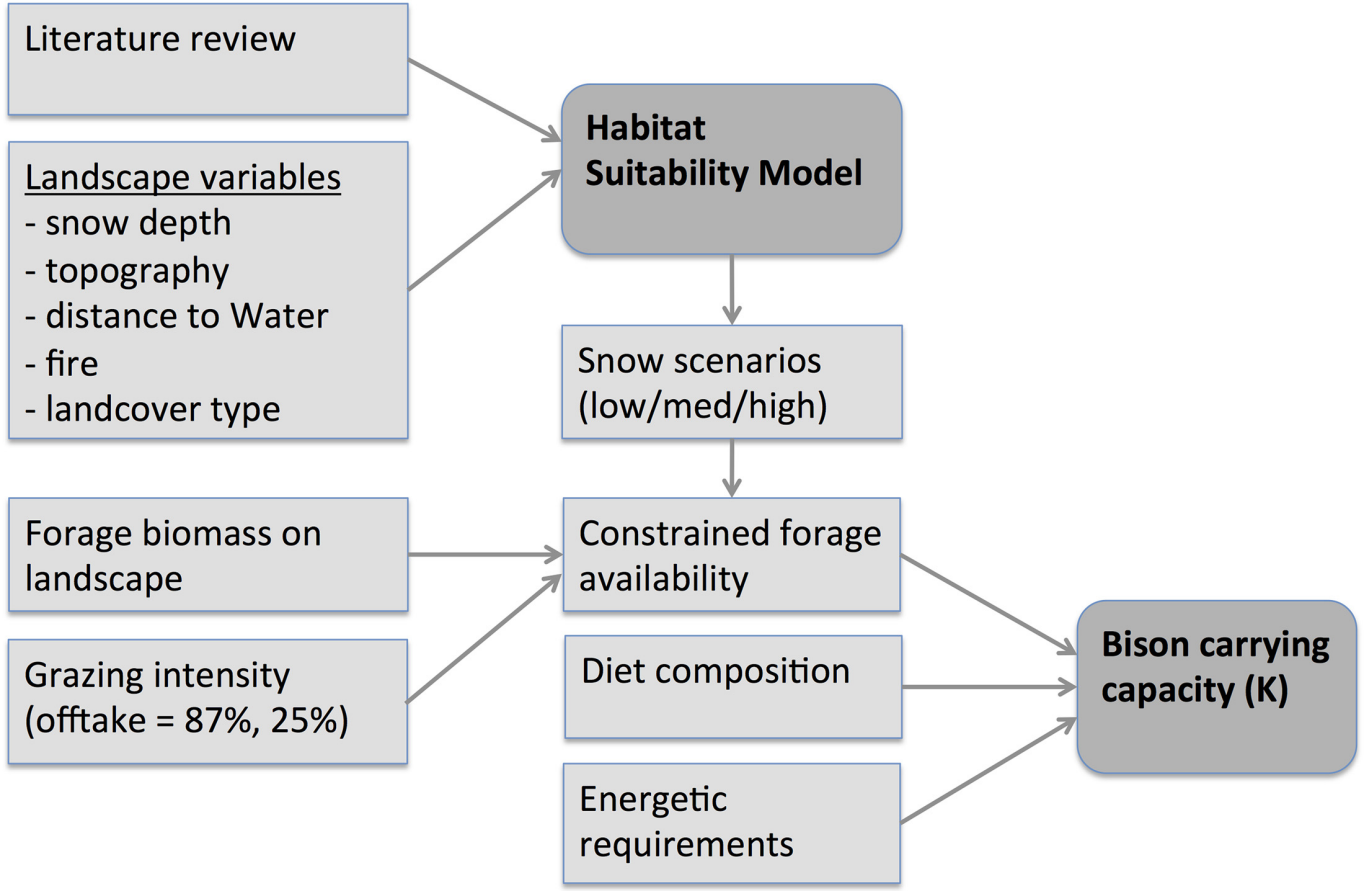
Conceptual diagram for development of a Habitat Suitability Index model and estimating carry capacity for potential plains bison (*Bison bison bison*) reintroduction.

The study area for evaluating potential bison habitat and population size was the eastern portion of Banff and was divided into 2 hypothetical bison reintroduction areas in accordance with the Parks Canada reintroduction plans [41,42]: primary (1390 km^2^) and secondary (1641 km^2^) reintroduction areas (Fig 1). Within the primary reintroduction area, we evaluated four hypothetical areas to help prioritize areas for potential reintroduction: Red deer (435 km^2^), Panther-Dormer (424 km^2^), Cascade (286 km^2^), and Fairholme (245 km^2^; Fig 1). Our HSI and carrying capacity models were developed in two main steps (Fig 2). We first developed a spatially explicit GIS-based HSI model based on variables that affect bison habitat selection in the literature. Second, we estimated carrying capacity by combining information on the distribution of forage biomass in the study area with different nutritional constraints and with the spatial constraint of habitat availability under different snow-depth scenarios using the HSI model.

### Habitat suitability index

We followed general guiding principles for developing HSI models [38,43] and based the model on factors known to limit bison foraging and distribution. We used previously published relationships to predict how bison habitat suitability varies with landscape variables. When many published relationships were available, we prioritized the empirical relationships from the study area(s) that most closely resembled our mountainous study area. When no relationship was available from a comparable area, we averaged all empirical relationships from the literature. Based on these variables for each raster cell (30 x 30 m), we estimated relative suitability ranging from 1 (unsuitable) to 10 (optimal suitability). Because of the importance of snow depth in limiting bison foraging and the unpredictability of annual snowfall, we developed 3 separate HSI models for 3 snowfall scenarios (low, medium, and high snow depth).

#### Literature review

We reviewed existing scientific literature for both plains and wood bison habitat selection in mountainous terrain (S1 Table). We also included papers from Prince Albert National Park, Saskatchewan, because they provided quantitative information on bison-snow interactions, whereas there were few specific studies of bison-snow interactions in montane systems. We focused on published studies that used landcover and GIS habitat modeling to understand bison habitat. Based on our literature review, we identified 5 landscape variables as potentially important for bison habitat selection in Banff: snow depth, topography, distance to water, fire, and landcover type. We summarize results from previous studies and describe how we developed the habitat suitability index (HSI) model, functions, and rankings for each variable, below. It is important to note that most empirical studies of bison habitat selection only radiocollared female bison (with the exception of [44]), therefore, our HSI model is focused on females.

#### Snow depth

**Fig 3:**
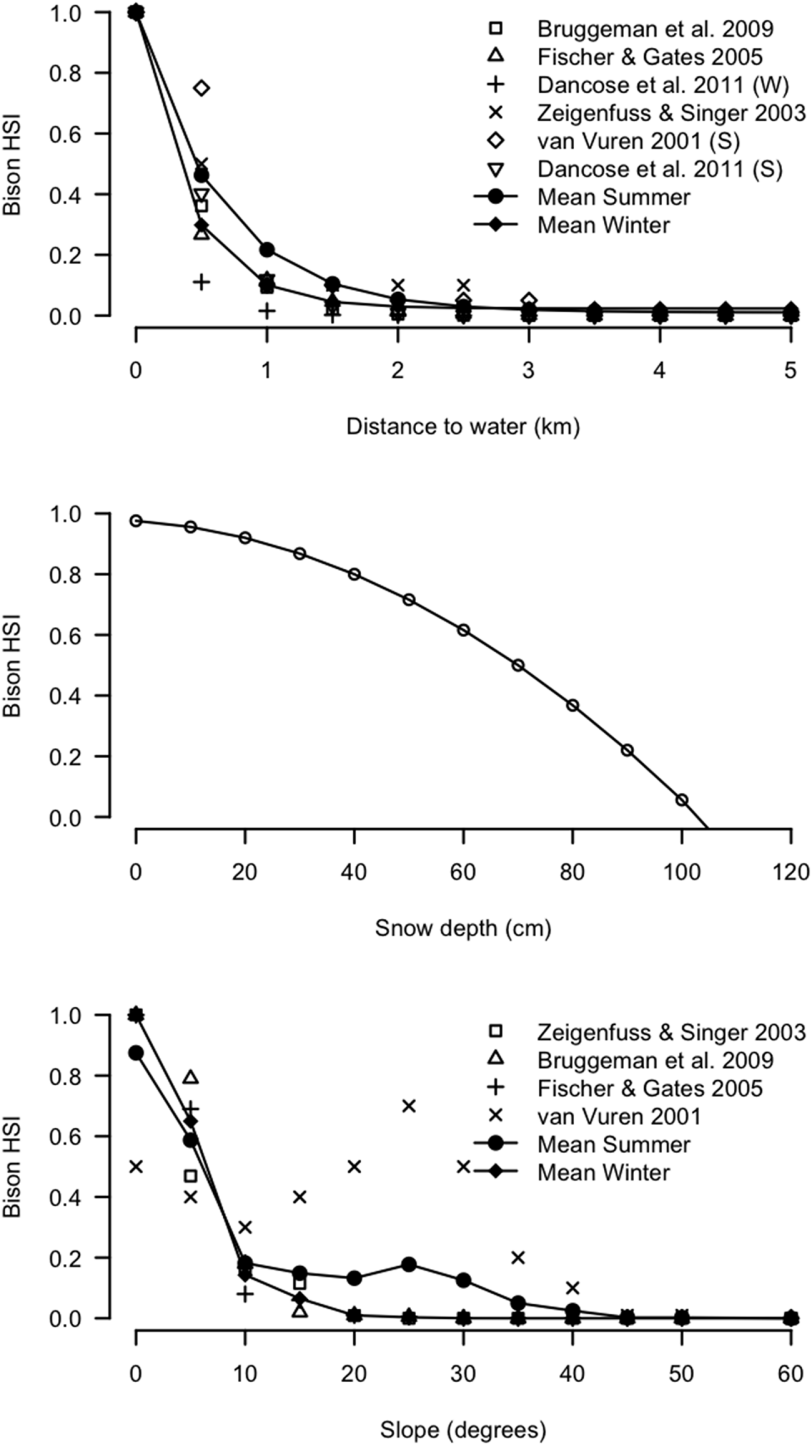
Potential plains bison (*Bison bison bison*) habitat suitability as a function of a) snow depth b) slope and c) distance to water during winter and summer in Banff National Park based on a literature review of previous studies.

Bison-snow relationships have been studied in Yellowstone and Prince Alberta National Parks. Although Bruggeman et al. [21] and Bjornlie and Garrot [45] related snow depth measured by Snow Water Equivalents (SWE) to bison movement, west Yellowstone National Park has geothermal features that creates high variability in snow-depth which allows bison to exploit snowless habitat; this may hinder extrapolation to areas of higher snow depth like Banff. We focused, therefore, on studies in Prince Albert National Park where bison more regularly encounter deeper snow [20,46]. Although Prince Albert is not mountainous like Banff, we assumed the relationship between snow depth and bison does not vary as much by topography. They estimated a model for bison density which predicted bison density = 1.20–0.03 DEPTH (cm), where the X intercept (i.e., where local bison density = 0) was 40 cm. Similarly, Fortin [46] and Fortin et al. [47] found that snow depth as measured by SWE, had large effect on winter travel in a slightly quadratic function (Fig 2 from Fortin [46]). They found both small and large groups of bison were similarly affected by snow depth such that the relative probability of use declined rapidly above 40 cm, and was essentially zero at 100 cm. For bison in Banff, therefore, we created a non-linear function relating bison HSI to snow depth, modeled after Fortin and Andruskiw’s [20] study (Fig 3a) where:

$$\text{Bison HSI }=\;1.0\;-\;0.00008{\text{ x snowdepth}}^{2}\;–\;0.0012\text{ x snowdepth }\left(\text{cm}\right)$$ (1)

To estimate snow depth, we used data from the Snow Data Assimilation System (SNOWDAS; [48]) from 2009–2012. To characterize effects of interannual variability of snow depth on habitat suitability, we selected snow depth data from an average, low, and high snow-depth winter to provide 3 potential habitat constraints for HSI modeling. Using the Banff Environment Canada weather station (Environment Canada, http://climate.weather.gc.ca/climateData/dailydata_e.html?StationID=27378) data available from 1996–present, we selected winter 2010 as the ‘low’ example winter, 2012 as the high winter, and 2011 as the average winter. For example, on March 16, 2010, snow depth ranged across Banff from 0–181 cm (mean = 78); in 2011 it ranged from 0–229 cm (mean = 119); in 2012, it ranged from 0–290 cm (mean = 159). To avoid selecting snow depths following a major snow event, we averaged daily SNODAS data for March, the peak month of winter snow accumulation.

#### Topography

Comparing bison selection for elevations across study areas is challenging because of the variation in the baseline elevation at valley bottom. As a result, we focused on summarizing bison selection for primarily slope and aspect across studies. During winter, bison in Jackson Hole, Wyoming [49], west Yellowstone [21] and the Yukon Territory (YT; [50]) strongly avoided steep slopes > 15 degrees slope. Using these 3 winter RSF-based studies, we developed an average bison slope HSI (Fig 3b) where:

Bison winter HSI = 1.0 for slopes 0–5°, 0.65 for slopes 5–10°, 0.15 for slopes 10–15°, 0.06 for slopes 15–20°, and 0.01 for slopes > 20° (2)

During summer, very few studies have compared slopes for bison habitat use, especially in mountainous study areas. The only published study showed, in contrast to summer, that bison used mid slopes in the Henry Mountains, Utah [51], favoring slopes of 30 degrees. However, in the Black Hills, South Dakota, there was little variation between bison habitat use in winter and summer [52]. For this reason, we averaged all 3 winter studies and the Van Vuren [51] summer habitat selection results to reflect broader summer selection for slopes following:

Bison summer HSI = 0.85 for slopes 0–5 degrees, 0.58 for slopes 5–10, 0.18 for slopes 10–15, 0.15 for slopes 15–20, 0.13 for slopes 20–25, 0.18 for slopes 25–30, 0.13 for slopes 30–35, 0.05 for slopes 35–40, and 0.01 for slopes > 40 (3)

Studies in Jackson Hole, Wyoming, and the Yukon showed that aspect affected bison habitat selection through its influence on snow accumulation in winter. During both early and late winter in Jackson Hole, bison showed strongest selection for southwest, flat, and northwest aspects, neutral selection for south and north aspects, and avoided aspects from northeast to southeast [49]. In the Yukon bison showed strongest selection for flat aspects, west and north facing [50]. Therefore, we modeled bison selection for aspect during winter following:

Bison HSI = 1.0 for flat aspect, 0.75 for southwest, west, or northwest, 0.5 for north or south, 0.25 for northeast, east, or southeast (4)

During summer, bison selection for aspect was assumed to be constant because of a lack of information in the literature. For both slope and aspect, we used digital elevation data (30-m resolution) obtained from Geogratis Canada. We used the Spatial Analyst extension for ArcGIS 9.3 to estimate slope and 9 aspect classes, including flat.

#### Distance to water

Water availability has been shown in previous studies to affect bison habitat suitability, where bison normally show strong selection for areas close to water. Studies in Yellowstone National Park [21,49], the Yukon [50] and Prince Alberta National Park [53] all provided quantitative results for selection as a function of distance to water, Van Vuren [51] and Dancose et al. [53] did so for bison selection during summer in the Henry Mountains and Prince Alberta National Park, respectively. We averaged these results to model bison HSI (Fig 3c) as a function of distance to water using an exponential model as follows:

$$\text{Bison winter HSI }=\;0.023\;+\;0.978\text{ x }0.079^{\text{Distance to water }\left(\text{km}\right)}$$ (5)

$$\text{Bison summer HSI }=\;1.01\text{ x }0.209^{\text{Distance to water }\left(\text{km}\right)}$$ (6)

In the study area, we calculated distance to water in GIS as the distance from the water landcover class in a model developed using LANDSAT imagery by McDermid [54] and modified for ungulates by Hebblewhite et al. [55].

#### Fire

The relatively few studies of bison habitat use of burned areas in mountainous terrain include: Alaska, Jackson Hole, Yellowstone National Park, Utah, and British Columbia [44,49,56,57]. Few of these studies reported quantitative selection or use, and when they did, it was mostly during winter. We developed a winter HSI based on previous quantitative studies for bison (Table 1 and S2 Table). We included 3 burn classes in the landcover model discussed in the subsequent section: burned-forest, burned-grassland and burned-shrubland. To do so, we updated the landcover model for fires developed in 2006 by Hebblewhite et al. [55] using updated 2012 fire polygon data from Banff (Parks Canada, unpublished data).

**Table 1:** Plains bison (*Bison bison bison*) habitat suitability (from 0, low to 1, high) for landcover types for winter and summer in Banff National Park, Alberta, Canada, based on standardized rankings for homologous landcover types from previous published studies on bison habitat use. See S2 and S3 Tables for more details.

Banff Landcover Variables	Summer Mean HSI	Rank	Winter Mean H.S.I	Rank
snow/ice	0.00	13	0.00	13
rock	0.01	12	0.05	12
open conifer	0.36	6	0.40	5
closed conifer	0.32	7	0.17	7
mixed forest	0.31	8	0.36	6
deciduous	0.27	10	0.60	3
herbaceous	0.66	1	0.87	2
alpine herbaceous	0.30	9	0.13	9
shrub	0.44	3	0.55	4
alpine shrub	0.25	11	0.13	9
burned forest	0.42	4	0.00	13
burned grassland	0.50	2	0.88	1
burned shrubland	0.40	5	0.13	9

#### Landcover types

Previous studies show that bison habitat selection is strongly influenced by landcover (see S1 Table). We used an existing landcover map derived from a supervised classification of LANDSAT at a spatial resolution of 30m2 [54,58] with modifications developed by Hebblewhite et al. [55] for elk in Banff, and updated to 2012 as described above. Landcover types included: closed conifer, moderate conifer, open conifer, shrublands, upland herbaceous, mixed forest, deciduous, water, rock/ice, alpine meadows and alpine shrublands. This landcover classification was expanded to include three burned vegetation types (forest, grassland, and shrub, see above). Alpine meadows and shrublands were delineated using an elevation cut of 2200m [39]. Using this classification, we translated landcover categories from previous studies into these landcover types for use in the bison HSI for Banff (Table 1, S2 and S3 Tables).

First, we compared published landcover categories from each individual study and assigned the most equivalent landcover type in Banff, if possible. Some landcover crosswalks were not possible either because they did not exist in Banff (e.g., prairie dog towns in the Black Hills, South Dakota; [59]) or because the Banff landcover model was not sufficiently detailed to contain a homologue (e.g., wetlands). Moderate conifer was assumed to be equivalent to closed conifer landcover types because no studies separated moderate conifer. Second, for each individual study, we ranked landcover from most (1) to least used or the most avoided (10). We then standardized the rankings for each study relative to the total number of ranks. To rescale standardized rankings such that high quality landcover types had a higher HSI we subtracted the standardized ranking from 1. Resulting HSI scores for each landcover type in winter and summer are shown in Table 1 with details of how we matched homologues in S2 and S3 Tables.

#### Final HSI model

To estimate bison habitat suitability during winter and summer, we combined eqs 1–6 with the landcover values in Table 1. We then evaluated the differences in the amount of available habitat between seasons by comparing HSI values between summer and winter. Based on importance of landcover types from the literature for summer bison habitat selection across studies (Table 1 and S1 Table), we weighted bison landcover during summer as twice as important as slope or distance to water. In contrast, in winter, we weighted both landcover and snow depth as twice as important as all other covariates in weighting the winter HSI model. The final equations are as follows:

Bison HSI Summer = 0.25 x Slope + 0.25 x Distance to Water + 0.50 x Landcover (7)

Bison HSI Winter  = 0.30 x Snow + 0.14 x Slope + 0.14 x Aspect + 0.14 xistance to  Water + 0.28 x Landcover (8)

To map the HSI models to compare habitat suitability among summer and the three different winter HSI models, as well as among areas, we divided continuous predictions of bison habitat suitability into 10 equally-sized bins.

Lastly, we evaluated the predictive capacity of our Bison HSI model using archaeological sites with bison remains (reported in [14] supplemented by unpublished data from B. Perry and G. Langemann, unpublished data). Because bison were extirpated prior to Park establishment, there were no other historical records. To validate models, we adopted a k-folds procedure [60] whereby the expected frequency of bison locations in each of 10-ranked categories of habitat suitability from 1 to 10 was compared to that expected based on the availability of bison habitat quality. We estimated expected proportions based on bison habitat within 1500m of roads and trails to account for the distribution of archaeological sites being similarly close to trails. We then calculated the Spearman rank correlation between the HSI values and the area-adjusted frequency of bison sites following [60]. If predicted bison habitat suitability explains observed bison archaeological sites, then the Spearman rank correlation should be positive and close to 1.0. We validated both the summer and winter-mean snow depth models using the archaeological sites because the seasonality of archaeological sites was unknown.

### Estimation of bison carry capacity

Our second goal was to estimate bison carrying capacity given the potential habitat available. We focused on winter under the assumption that this was the season that sets the upper limit on bison population size [28,29]. First, we estimated available forage biomass (Fig 2), then we constrained availability based on grazing intensity, HSI, and the three snow-depth scenarios. Next we combined this constrained forage availability with previous studies on bison diet composition and energetic requirements to estimate carry capacity. Our approach did not account for any limits on forage quality for estimating winter carrying capacity, e.g., [32], rather, we assumed forage quality of graminoids is nearly constant through the winter and forbs are largely unavailable at this time of year.

#### Available forage biomass

We used estimates of forage biomass (productivity) in kg/ha in August reported by [55] estimated using ground-based biomass vegetation sampling of ~1000 vegetation plots from 2002–2005 and extrapolated to all landcover types (updated to 2012, S4 Table). This model was a predictive model of forage biomass based on spatial covariates including landcover, topography, and remotely-sensed measures of forage productivity, the Normalized Difference Vegetation Index (NDVI) measured by the MODIS satellite (see [51] for more details). NDVI is correlated to both forage biomass and quality in open herbaceous landcovers during the growing season [61]. This approach assumed no forage depletion over the winter, which is a reasonable assumption given the extremely low winter density of elk in the study area. Because knowledge of bison diet composition in Banff at the plant-species level is unknown, we used dry matter intake rates of plant-forage class (shrub, graminoid, forbs). Forb biomass was assumed to be only 1% of summer forb biomass across all landcover types through the retention of dried stalks of certain forb species such as *Artemesia* spp., *Geum* spp., etc., that occasionally remain through the winter and that were reported to be consumed by bison at an extremely low percentage [59]. Graminoid biomass included grasses and sedges, but was not separated by landcover type. For shrub species, we used known elk forage species only (*Salix* spp., *Potentilla* spp., *etc*.), i.e., we did not include biomass of other shrubs that are likely consumed by bison (e.g., excluding Azalea, Alder, *Shepherdia canadensis*, etc.). This omission is not likely to significantly affect final estimates because we estimated bison diet composition to be only ~5% shrubs (see next section).

#### Constraining forage availability

Many previous attempts to estimate nutritional-based carrying capacity for ungulates have been criticized by unrealistically assuming all forage biomass is available to ungulates [28]. Using estimates of K on seasonal intake rates (kg/day/bison) and total standing crop of forage biomass (kg/ha) to calculate density of bison/ha assumes 100% forage consumption during the season in every single landcover type that is available to bison. Instead, we used three constraints to make this nutritional-based carrying capacity more realistic. First, we used previously published estimates of average forage offtake to constrain the maximum forage possible to consume. Turner et al. [34] note, for example, that because bison paw through snow or use head swings to move snow, the snow forms a hard crust that then renders the forage unavailable until after a significant warming event. They used field trials to estimate a refuge for plants of 13% of the standing forage biomass, equivalent to a maximum offtake of 87%. At the lower end of published studies, Kuzyk et al. [29] reported results of carrying capacity estimates for 4 ungulates species in Elk Island National Park using 7–15% forage use for graminoids and forbs, and 60% for shrubs, based on empirical observations in that system. Similarly, Sachro et al. [62] used a grazing intensity of 25% to estimate elk nutritional carrying capacity in Banff. To characterize this uncertainty, we chose 87% and 25% scenarios to constrain forage offtake across landcover types.

Second, we used the Habitat Suitability Index (HSI) model to constrain bison accessibility to different landcover and terrain types following Beck et al. [36] who similarly used an HSI as a weighting factor on maximum predicted bison population size (Fig 2). We used this approach with the two grazing intensity scenarios outlined above (87% and 25%). Finally, by using our different HSI models that already integrate snow depth as a habitat suitability factor (low, medium and high), we explored the effect of snow on potential nutritional-based carrying capacity estimates for bison. We used the relative changes in the mean HSI values to reduce estimated carrying capacity for bison in the study area from the constrained forage availability (Fig 2).

#### Diet composition for bison during winter

Across studies, forage composition of bison in winter was dominated by graminoids. Coughenour [33] reported that plains bison diet in Yellowstone National Park during winter constituted 95% graminoids and 5% shrubs. Kuzyk et al. [29] reported 96% graminoids and 4% shrubs for bison (both wood and plains bison) in Elk Island National Park. In the MacKenzie Wood Bison Sanctuary, Northwest Territories, Larter [63] reported 96.1–98.8% diet composition of graminoids during winter, with the remainder being shrub biomass. In the Black Hills, South Dakota, graminoids constituted 93% of winter diet, shrubs 5.2% of the diet, and forbs 1.8% of the diet [59]. Averaging these studies, we estimated bison diet composition during winter to be 95% graminoids, 4% shrubs and 1% (trace) forbs.

#### Energetic requirements for bison

Winter bison intake rates in kg/day adjusted for age structure were averaged across previous studies that similarly calculated winter carrying capacity [29,32–34,59,64]; see S5 Table for summary). We averaged age structures from Yellowstone and the Black Hills, South Dakota for an assumed simplified age structure of 30% adult bulls weighing 800 kg, 50% adult females weighing 440 kg, and 20% calves weighing 220 kg (Table 2). Note that we weighted adult body mass for yearling age structure. Using these body masses, we then averaged intake rates across studies to 2.5% of body weight for adult bull bison, 2.75% for adult female bison, and 3% for juvenile bison (Table 2). Finally, we used linear programming with Microsoft Excel’s Solver to solve for the linear solution that achieved a diet intake of 95% graminoids, 4% shrubs and 1% forbs using available forage biomass in each forage-class component across landcover types. Because of the dominant role of graminoid biomass, when shrub or forb biomass was limiting, this essentially equated to selecting the minimum potential population estimate across these three different forage classes. Bison numbers were rounded down to the nearest whole number and reported in numbers and density.

**Table 2:** Potential energetic requirements for plains bison (*Bison bison bison*) in Banff National Park, as a function of age structure, body mass, and % daily intake rates available in literature.

Age Class	Proportion of population	Body Mass (kg)	% Daily intake rate	Daily intake rate (kg/day)
Adult male	0.3	800	2.5	20
Adult female	0.5	440	2.75	12.1
Juvenile	0.2	220	3	6.6
Total weighted intake rate for population (kg/day):				13.4
Total winter forage (181 days) intake (kg/winter):				2,419.9
Total forb intake rate (kg/winter):				24.2
Total graminoid intake rate (kg/winter):				2,298.9
Total shrub intake rate (kg/winter):				96.8

## Results

### Bison habitat suitability

Our bison HSI models predicted the independent archaeological observations of bison (n = 13) in Banff well, despite our small sample size. In summer, the Spearman correlation between HSI value and area-adjusted frequency of bison sites was rS = 0.614 (p = 0.059), with 84.6% of bison sites occurring in 15.7% of the landscape where HSI > 7. During winter, the Spearman correlation between HSI value and area-adjusted frequency of bison sites was weaker at rS = 0.533 (p = 0.174), with 100% of bison sites occurring in 32.6% of the landscape where HSI > 4.

**Fig 4:**
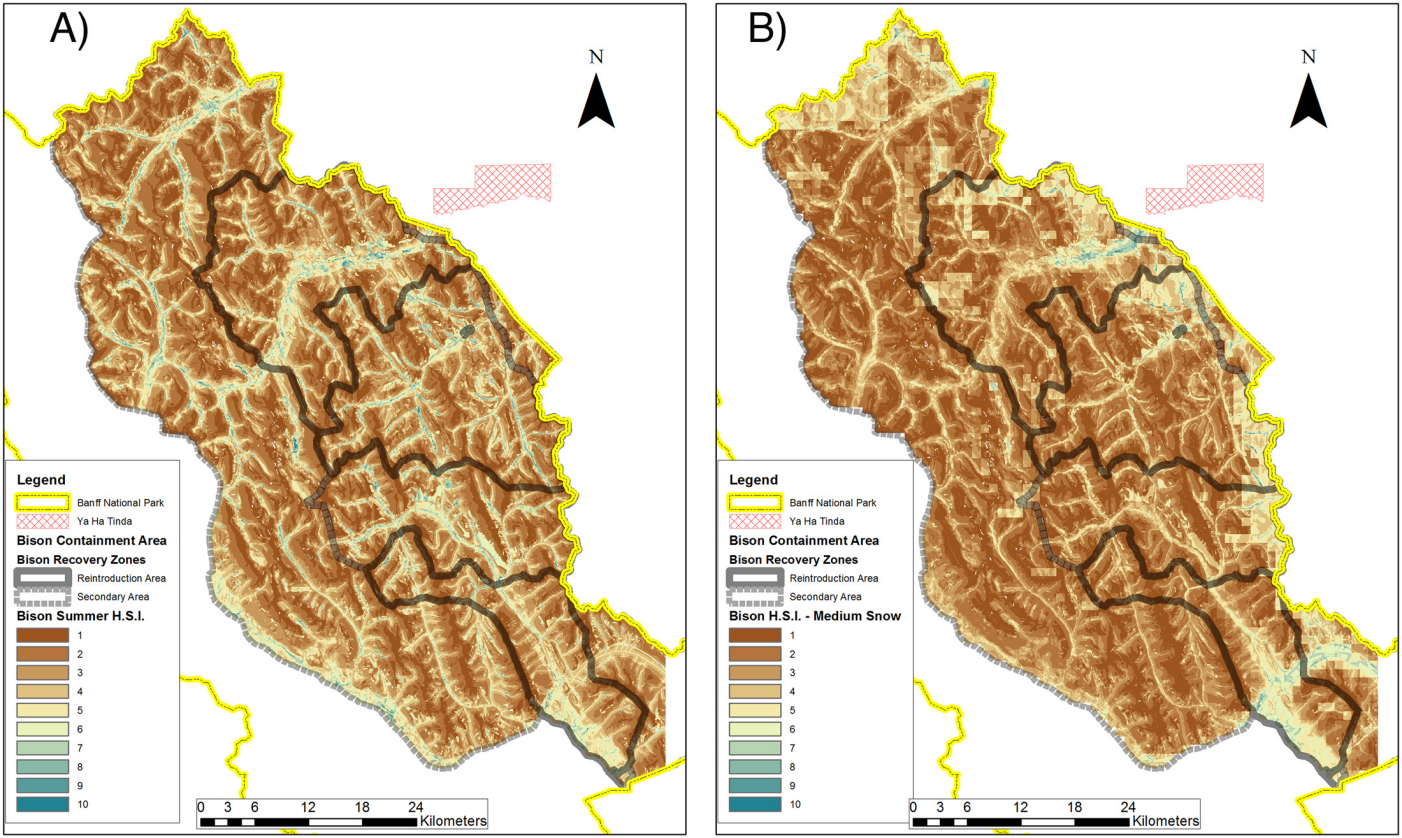
Predicted plains bison (*Bison bison bison*) Habitat Suitability Index (HSI, low of 1 to a high of 10) in the primary reintroduction and secondary areas of Banff National Park. Left panel is HSI for summer, right panel for a medium snow-depth winter (using snow data from 2011).

Overall, winter habitat suitability was lower than summer habitat suitability by about 50% (Table 3). Regardless of season, the highest suitability habitats for bison were concentrated in valley bottoms, mid-mountain slopes close to water, and areas with substantial burns (Fig 4). Visually, the Fairholme area had the highest apparent habitat suitability, and this was supported by comparison of mean HSI rank for each area shown in Table 3. The mean HSI value in summer was 5.7 in the entire primary reintroduction area and 5.9 in the secondary area. HSI values were 6.3 in the Fairholme, 5.7 in the Panther-Dormer, 5.4 in the Red Deer, and 5.3 in the Cascade areas. The difference in potential habitat suitability between areas in the summer was a function of the availability of burned forests and grasslands, upland herbaceous, and areas close to water in mid-slopes as driven by the HSI equation.

**Table 3:** Mean Habitat Suitability Index (HSI, low of 1 to a high of 10) values for plains bison (*Bison bison bison*) habitat during summer, and during 3 different winter scenarios of low, medium and high snow depths in Banff National Park.

	Summer HSI	Winter HSI		
Area		Low Snow	Med. Snow	High Snow
Primary Areas	5.7	3.9	2.9	2.7
Panther-Dormer	5.3	3.4	2.6	2.6
Red Deer	5.4	3.7	3.0	2.7
Cascade	5.7	4.0	2.8	2.7
Fairholme	6.3	4.6	3.1	2.9
Secondary Area	5.9	3.1	2.6	2.5

In winter, bison habitat suitability was predicted to be the highest under low snow scenarios in the Fairholme area, followed by the Panther-Dormer, Red Deer and Cascade (Table 3). As modeled snow depth increased, the Fairholme remained the highest-ranked area for average bison habitat suitability and the ranking between areas generally stayed the same with Panther-Dormer and Red Deer being more or less equivalent under the high snow depth scenario. For example, in the medium snow depth scenario, the mean HSI for the primary reintroduction area was 2.9, in the secondary area 2.6, and was 3.1 in the Fairholme, 3.0 in the Red Deer, 2.8 in the Panther-Dormer, and 2.6 in the Cascade (Table 3).

### Bison carrying capacity

Potential densities estimated with our nutritional carrying capacity model demonstrated that even with the reduced habitat quality for bison in winter, there could be sufficient habitat to support a large bison population year round. Building on our HSI model, under the maximum grazing intensity scenario (87% offtake), potential winter bison population estimates for the entire primary reintroduction and secondary areas ranged from 4520–6250 bison, or 1.5–2.1 bison/km^2^ (Table 4). In the primary reintroduction area only, maximum bison population size was projected at 2520–3690 or 1.8–2.7 bison/km^2^. Highest estimates assumed 87% forage utilization and low snow during winter. Under the mean snow depth scenario, maximum bison population size in both the primary reintroduction and secondary areas was 4790 potential bison or 1.6 bison/km^2^ (Table 4). Just in the primary reintroduction area, under mean snow depths, there were 2690 potential bison or 1.9 bison/km^2^. Within the primary reintroduction areas, population size was projected to be highest in the Panther-Dormer area with 1400 bison, followed by the Fairholme with 560, 560 in the Red deer and 440 in the Cascade. In terms of density, the Panther-Dormer also had the highest density at 3.2 bison/km^2^, 2.3 in the Fairholme, 1.9 in the Red deer and 1.6 in the Cascade.

**Table 4:** Potential plains bison (*Bison bison bison*) density (bison/km^2^) during winter in primary reintroduction and secondary areas of Banff National Park under the two grazing intensities (87% and 25% offtake) and three winter severity scenarios.

Snow-depth:	Low		Medium		High	
Grazing Intensity:	87%	25%	87%	25%	87%	25%
Primary Areas	2.7	0.7	1.9	0.5	1.8	0.5
Panther-Dormer	4.2	1.0	3.2	0.8	3.2	0.8
Red Deer	2.4	0.6	1.9	0.5	1.7	0.4
Cascade	2.3	0.6	1.6	0.4	1.5	0.4
Fairholme	3.5	0.9	2.3	0.6	2.2	0.5
Secondary Area	1.6	0.5	1.3	0.4	1.2	0.4
**Total**	2.1	0.6	1.6	0.4	1.5	0.4

Under the more realistic 25% grazing scenario, potential bison population estimates for the entire primary reintroduction and secondary areas ranged from 1210–1660, or 0.4–0.6 bison/km^2^ (Table 4). In the primary reintroduction area only, maximum bison population size was projected at 630–920 or 0.5–0.7 bison/km^2^. High estimates assumed 25% forage utilization and low snow during winter. Under the mean snow depth scenario, maximum bison population size was 1280 or 0.4 bison/km^2^ within both the primary reintroduction and secondary areas, or 670 or 0.5 bison/km^2^ just within the primary reintroduction area. Within the primary reintroduction area, projected population size was highest in the Panther-Dormer area with 350 bison, followed by 140 in the Fairholme, 140 in the Red deer and 110 in the Cascade. In terms of density, the Panther-Dormer also had the highest density at 0.8 bison/km^2^, compared to 0.6 in the Fairholme, 0.5 in the Red deer and 0.4 in the Cascade (Table 4).

## Discussion

Based on our habitat suitability model and estimates of nutritional carrying capacity, there appears to be sufficient habitat of high-enough quality to support a relatively large population of plains bison year-round in Banff that could significantly contribute to improving their global conservation status. Despite evidence for a reduction in habitat suitability during winter by approximately 50%, the estimated winter population appears sufficiently large to support a greater population size than most other extant populations in North America [13]. Much of Banff’s subalpine and alpine regions, however, are not predicted to be functional bison habitat during winter, when bison habitat is limited to lower-elevation montane and subalpine areas in the Red Deer, Panther, Cascade and Bow valleys. This is because of the combination of favored landcover types, as well as snow depths that increase at higher elevation and bison responses to topography. Moreover, bison habitat suitability was strongly positively influenced by fire during both seasons, and especially in the winter when prescribed and natural fires in low elevation winter ranges contributed significantly to bison habitat suitability, as evidenced in empirical studies [44,49,56,57]. Given the paucity of summer RSF studies on bison and the demonstrated use of steep areas in Utah by the Henry mountains population [51], there remains uncertainty about bison use of steep terrain, especially in summer. Assuming that bison only use flat terrain in summer may result in an underestimation of bison distribution and thus movements in summer. Although water is not likely to be as important for bison in Banff compared to more arid areas such as Utah, snow is likely to have a strong effect on both bison habitat selection and carrying capacity. There is agreement among studies regarding the importance of snow for limiting bison habitat suitability (see methods). The striking difference between bison HSI models for summer and winter (compare Fig 4a and 4b) implies the importance of considering both seasons when predicting bison distribution and carrying capacity. Because of deep snow in many areas in Banff, bison are likely to use mainly valley bottoms in winter, and may only expand up to mid slopes during summer. In high snow-depth winters, higher-quality habitat is restricted to areas of low snow accumulation, such as the Red Deer and Bow valleys and far eastern slopes. There was also consistency between previous studies in selection for grasslands, shrub lands, and the strong avoidance of coniferous forests. Despite a few studies that showed minimal effects of burns on bison habitat selection, a growing number of studies clearly demonstrate that burns are important in improving bison habitat suitability [44,49,56,57]. Given that burns enhance green forage biomass and reduce standing dead biomass, especially in grasslands, quantifying bison use of burns post reintroduction will be important in both winter and summer.

Our estimated bison density ranged from 0.5 bison/km^2^ under the most conservative scenario to 5 bison/km^2^, reflecting the range of possible grazing intensity and snow depth conditions in our models. These bison densities, during winter, support the prediction that Banff will be able to sustain year-round bison populations. These bison population estimates are quite similar to others reported in the literature. In Yellowstone National Park the potential bison carrying capacity was reported to be 1.76 bison/km^2^ [18,33], similar to estimates from our 25% grazing scenario. We believe the most realistic scenario evaluated for projecting potential bison population size and density within the Banff reintroduction area is the 25% grazing intensity scenario under mean snow depth conditions. This equates to 670 bison (0.5 bison/km^2^) within the primary reintroduction area, another 610 potential bison (0.4 bison/km^2^) in the secondary area, for a total projected population size of 1280 bison (0.4 bison/km^2^) under mean snow depth conditions (Table 4). Within the primary reintroduction area, the highest numbers of bison and population density are projected to occur within the Panther-Dormer area with 350 bison (0.8 bison/km^2^), followed by the Fairholme with 140 bison (0.6 bison/km^2^), then the Red Deer with 138 bison (0.5 bison/km^2^), and lastly, the Cascade with 110 bison (0.4 bison/km^2^).

Based on our habitat suitability models, the Fairholme area had higher predicted habitat quality across seasons and across winter-severity scenarios, but a smaller area. Combining the results of the habitat suitability with our carrying capacity model, on the other hand, suggests that there is higher capacity for bison in the Panther-Dormer area, followed second by the Fairholme. Although both models used landcover types in their formulation, this discrepancy arose from spatially explicit differences between areas in on-the-ground estimates of forage production and availability within the landcover types. These two results from these analyses, potential habitat quality and potential population size, can be used to prioritize bison reintroduction strategies. Considering both results, the Panther-Dormer area could be ranked first for consideration for potential bison reintroduction, followed second by the Fairholme area. In part, this analysis informed the more recent proposal to begin bison reintroduction in the Panther-Dormer area [41]. Regardless of how potential bison reintroduction plans or management areas may change, our spatially-explicit results will allow managers to evaluate potential bison habitat under different scenarios or management areas.

Our 25% grazing utilization scenario however, likely overestimates bison population size because we did not account for nutritional or quality constraints on bison carrying capacity [28]. In Wood Buffalo National Park, Hamilton [32] compared scenarios of maximum forage biomass to a nutritional constraint-based model. Under no constraints, the bison population size was only limited by available biomass of forage; Hamilton [32] predicted ~ 33,000 bison, or > 22 bison/km^2^. Under more realistic scenarios of constrained forage species, estimates were 11,000 bison and 7–8 bison/km^2^. Finally, under nutritionally-constrained models that included information about diet preference and preferred forage quality, density estimates were 7 bison/km^2^ and ~ 10,000. Hamilton [32] showed that observed numbers of bison in Wood Buffalo National Park more closely corresponded to those predicted under these constrained models, especially in the Hay Camp area, but not the Peace-Athabasca Delta area. This suggested that other factors besides food alone were capable of limiting bison numbers in Wood Buffalo National Park. Despite the lack of nutritional constraints we could use in this model for Banff because of a lack of data on bison diet preference, comparing Hamilton’s [32] estimates with ours supports the interpretation that the 25% low intensity grazing scenario was close in magnitude to the difference between Hamilton’s [32] scenarios when including all forage species or just preferred forage species. Hence we recommend using the 25% low intensity scenario for projecting potential bison numbers in Banff.

Our carrying capacity assessment used similar methods to those recently developed for bison throughout North America [18,33,49,64]. This bottom-up perspective makes two simplifying assumptions; first, that predation by either humans or native carnivores will have no effect on the distribution of potential bison habitat or on carrying capacity. In Wood Buffalo, Prince Albert, and Yellowstone National Parks, for example, wolves are an important predator of bison [47,65] and foraging and habitat-selection choices may vary in the presence of wolves [47]. In areas of relatively low human activity, such as Wood Buffalo National Park, bison frequently flee human foot traffic [20]. Most of the proposed reintroduction area in Banff has very low human activity, and vehicle traffic only occurs in the southernmost portion of the area. Therefore, human effects on bison habitat selection may apply primarily in the southern end of the study area, but predators such as wolves occur throughout the proposed reintroduction area and affect habitat selection of another large herbivore, elk [66], and so may also constrain bison habitat availability. Regardless, all such bottom-up approaches also ignore important socioeconomic constraints to potential population size, and indeed, habitat-based carrying capacity should not be considered as a recovery target. Social carrying capacity, potential ecological impacts on other species, and other socioeconomic constraints often restrict management goals for bison and other species [67]. Thus, these nutritional carrying capacity estimates should only be considered as a valuable frame of reference on the theoretical, nutritional potential to support bison in the study area.

One limitation of this study is that we did not assess bison habitat outside of Banff. This was largely because of the paucity of information on relevant covariates in adjacent areas. The overall question we address, however, is whether there could be sufficient habitat for year-round bison in Banff. It is necessary to first address this question to understand if bison reintroduction could be feasible in Banff alone (i.e., not including expansion into the province of Alberta). As such, the initial goal of the Banff bison reintroduction plan [42] has been to retain bison within the park, which we focus on here. Nonetheless, bison habitat quality in surrounding areas is highly relevant and indeed, expected to be higher in some areas outside the park. The Ya Ha Tinda Ranch to the east of Banff (Figs 1 and 4), for example, is characterized by low-elevation montane grasslands with very low winter snow fall and is traditional winter range for elk [68]. Other bison populations roam across boundaries of Yellowstone [69], Wood Buffalo [70], and Prince Albert [46] National Parks. The frequency and extent of their seasonal movements vary depending on bison density, climate (*i*.*e*., snow depth), forage availability [18,37], and relative habitat suitability differential between areas inside protected areas and adjacent to. We found a ~50% reduction in habitat quality inside Banff during winter. In extreme snow years, therefore, it may be important to assess and understand habitat quality outside the park and to predict potential habitat selection and movement behavior of bison in surrounding areas. Bison in Yellowstone and Prince Albert National Parks have demonstrated the need for interagency cooperation and communication to deal with bison on both sides of park borders. Although we do not explicitly identify bison habitat outside of Banff, recovery planning will benefit from future work to assess the attractiveness of surrounding habitats for bison and development of effective strategies to manage potential transboundary movements.

If bison reintroduction proceeds in Banff, we make the following recommendations to test our bison habitat suitability model and improve estimations of carry capacity. Firstly, following their release, bison diet composition, diet preference, and forage quality should be monitored to develop forage-quality constraints on models of potential population carrying capacity [32]. Given recent success in validating bison habitat in Prince Albert and Grasslands National Parks by energy-based bison habitat models that include estimates of quality [71], this should be a priority for understanding bottom-up constraints on potential bison population size. Secondly, the SNODAS model we used for snow depth should be validated in Banff, not only for bison habitat prediction but also for other winter-wildlife and climate applications. Bison habitat selection and use should be monitored following reintroduction to validate, test and refine this habitat suitability model. Bison-habitat relationships with respect to burns should also be a priority for future research, due to their potential importance for bison. Once bison expand into areas with higher levels of human use, the interactions with human features (roads, trails) and human activity (tourism) should be investigated to understand the potential for these activities to enhance or diminish bison habitat in Banff. Finally, interspecific effects of predators or other ungulates on bison habitat should be monitored following reintroduction. Here we focused only on nutritional habitat for bison, and ignored relationships between bison densities and intraspecific interactions through herbivory, riparian impacts, and impacts of potential management efforts to contain bison in Banff (e.g., fencing). Evaluating these additional relationships was outside the scope of the present study, but future recovery efforts in Banff (and other bison populations) must address these additional considerations.

In conclusion, successful reintroduction of plains bison to Banff could represent a significant step for the global conservation for this iconic species and restore the functional ecological role of this large grazer in the park. Our research confirms that despite seasonal reduction in habitat for bison in winter, winter population sizes of bison could be expected to be sufficiently large to significantly contribute to global bison conservation. Even under our 25% grazing intensity scenario, the potential population size of plains bison in Banff could be in the top 20% of sizes of free-ranging bison populations in North America, a significant conservation achievement [13]. This is consistent with Parks Canada’s mandate to improve ecological integrity of national parks and may eventually contribute an additional subpopulation of wild plains bison in Canada. Most bison in North America are intensively and artificially managed for production or to control numbers, rather than limited by natural factors such as predators, weather and competition for mates [6]. Within Banff, bison may eventually be able to expand in range and numbers, and exist under natural selective factors within Banff. Reintroduction efforts such as this one hold much promise for contributing to long-term conservation of wild bison and restoration of this keystone species over large landscapes. Whether or not plains bison increase to sufficient ecological densities to restore their keystone role through grazing and interspecific interactions is less certain in Montane landscapes [72], but given that the potential habitat we identified could support one of the larger wild bison populations, should reintroduction proceed, there will be an excellent opportunity to test the ecological role of bison. Regardless of their potential ecological role, our approach to identify potential habitat and nutritional carrying capacity will be useful for reintroductions of other large herbivores to formerly occupied portions of their range.

## Supporting Information

S1 Table Summary of quantitative plains bison (*Bison bison*) habitat use and selection studies in the literature from which our HSI bison model was developed for Banff National Park. (DOCX)

S2 Table Plains bison (*Bison bison bison*) winter landcover suitability (from 0, low to 1, high) for Banff National Park. Scores based on standardized rankings for homologous landcover types from previous published studies on bison winter habitat use. Dashes indicate no homologous landcover type was studied.
(DOCX)

S3 Table Plains bison (*Bison bison bison*) summer landcover suitability (from 0, low to 1, high) for Banff National Park. Scores based on standardized rankings for homologous landcover types from previous published studies on bison summer habitat use. Dashes indicate no homologous landcover type was studied.
(DOCX)

S4 Table Forage biomass (kg/ha) as a function of landcover type, and proportion of landcover type, in the primary and secondary reintroduction areas of Banff National Park, separated into forb, graminoid and shrub biomass. See Hebblewhite et al. [55] for more details.
(DOCX)

S5 Table Summary of literature values for plains bison (*Bison bison*) intake rate estimation including reported age and sex structure that were used for bison carry capacity estimation for Banff National Park. (DOCX)
