# Supplementary material for: Assessing Potential Habitat and Carrying Capacity for Reintroduction of Plains Bison (Bison bison bison) in Banff National Park
Source: PLoS One. 2016 Feb 24;11(2):e0150065. doi: 10.1371/journal.pone.0150065 (PMC4765961; doi:10.1371/journal.pone.0150065)
Supplement: S1 Table — (DOCX) [file pone.0150065.s001.docx]

**Supporting Information**

**S1 Table. Summary of quantitative bison (*Bison bison*) habitat use and selection studies in the literature from which our HSI bison model was developed for Banff National Park.**

| **Study** | **Title** | **Sub-species** | **Study Area** | **Methods** | **Season** | **Covariates** |
| --- | --- | --- | --- | --- | --- | --- |
| Bruggeman et al. [1] also Bruggeman et al. [2] | Effects of Snow and Landscape Attributes on Bison Winter Travel Patterns and Habitat Use | Plains bison | Hayden and Pelican Valley | 30 GPS collars (15 from 2003-2004, redeployed in Nov 2004 ~2006), fix every 30 minutes from 7.00AM-7.00 PM, third order matched case control. Note – nothing about nighttime bison locations. | Winter (Nov-Apr) | SWE, Topography: average slope (SLOPE), slope heterogeneity (SLOPE het); habitat: meadow, burned forest, unburned forest, geothermal, or other; distances to stream (DISTstream), burned forest (DISTburn), unburned forest (DISTunburn), foraging area (DISTforage), and road (DISTroad) |
| Zeigenfuss and Singer [3] | Ecology of Native Ungulates in the Jackson Valley Habitat selection, interactions with domestic livestock, and effects of herbivory on grassland and willow communities | Plains bison | Jackson Valley | Aerial survey data from Jackson Valley, 1997-1999; used locations versus equal amount random locations within study area; removed used locations within feeding grounds; RSF; habitat preference | Early winter (Nov-Dec); late winter (Jan-Apr) | Vegetation type, distance to water (rivers and lakes), SWE, elevation, slope, aspect, hunting (elk hunting areas), and burns (<5 yrs) |
| **Study** | **Title** | **Sub-species** | **Study Area** | **Methods** | **Season** | **Covariates/Results** |
| Fischer and Gates [4] | Competition potential between sympatric woodland caribou and wood bison in woodland caribou and wood bison in southwestern Yukon, Canada | Wood bison | Aishihik Lake area in the southwestern Yukon | late winter aerial surevey data 1988-2001, compared overlap at 4 scales: slection and overlap at landscape scale, smaler scale "range overlap scale" of wood bison and woodland caribou | Winter | Elevation, slope, aspect, terrain ruggedness, and distance to permanent water bodies (i.e., major lakes and rivers) |
| Van Vuren [5] | Spatial relations of American bison (Bison bison) and domestic cattle in a montane environment | Reintroduced plains bison | SW Utah Henry Mtns. (375 ha basin) | daytime observation of foraging locations from vantage points; measured USE (not selection) | Summer | Slope, distance (horizontal and vertical) to water, elevation |
| Dancose et al. [6] | Mechanisms of functional connectivity: the case of free-ranging bison in a forest landscape | Plains bison | Prince Albert National Park | 23 GPS Collar on female bison between March 2005 and August 2008 .GPS locations 3 hrs 5 days a week and every hr 2 days a week | All four seasons | Meadow area, average plant biomass, squared biomass , presence of a water body within the meadow (e.g., a pond) and, for the winter model, the average SWE of the meadow. |
| **Study** | **Title** | **Sub-species** | **Study Area** | **Methods** | **Season** | **Covariates/Results** |
| Duchense et al. [7] | Mixed conditional logistic regression for habitat selection studies | Plains bison | Prince Albert National Park | 2005-2008; 24 females GPS collared, each used paired with 10 random within 1.6 km radius, fixed- and mixed c-logit | Spring (Mar-May) | Land-cover types: meadow (incl areas near lakes and rivers dominated by grasses, forbes, sedges), riparian (shrubs near streams and rivers), forest (decidous, conifer and mixed), water, road (incl areas <15m from human made trail or road), farmland; forest = ref. category |
| Leverkus [8] | Seasonal Range use by Wood Bison in British Columbia | Wood bison | Alaska Highway in NE BC; Nordquist and Ettithun herds | 10 GPS collars over 2 years, 1 location/hour fix rate. Aerial surveys | Feb 2009 - winter 2012 | Basic patterns of home range size (6 – 35 km^2^), basic patterns of habitat use of burned habitats. However, insufficient information is presented to be able to estimate selection strength for burns. |
| Keller [9] | Factors affecting spatial and temporal dynamics of an ungulate assemblage in the Black Hills, SD | Plains  bison | Custer State Park (Black Hills), SD | Seasonal RUF (resource utilization function) of 25 female VHF collared bison from Oct 2005 – Aug 2008 | All four seasons. (We include fall and spring with summer) | Strongly avoided burned forests, selected shrubs, mixed grass prairie and upland shrublands. Avoided areas far from water. |
| Larter [10] | Diet and Habitat selection of an erupting Wood bison population | Wood bison | McKenzie Wood Bison Sanctuary | Selection ratio of aerial locations during summer | Summer (used Fischer and Gates for winter) | Grasslands and shrub lands selected the most, deciduous forests intermediate, mixed forest neutral, avoided coniferous forests. |
| **Study** | **Title** | **Sub-species** | **Study Area** | **Methods** | **Season** | **Covariates/Results** |
| Ranglack et al. 2015 | Habitat Selection by Free-Ranging Bison in a Mixed Grazing System on  Public Land | Plains Bison | Henry Mountains, Utah | 2011 – 2013, 25 GPS collared females, 20 males, Resource Selection Function | Summer and Winter | Male and female bison differed between fall and winter in habitat selection, but not during summer. Patches of grassland, whether naturally occurring or created through burning or mechanical  treatments, were favored regardless of their distance to water. |

References

1. Bruggeman JE, Garrott RA, White PJ, Bjornlie DD, Watson FGR, et al. (2009) Effects of snow and landscape attributes on Bison winter travel patterns and habitat use. In: Garrott RA, White PJ, Watson FGR, editors. The ecology of large mammals in central Yellowstone: sixteen years of integrated field studies. San Diego, CA: Academic Press. pp. 623 - 647.

2. Bruggeman JE, Garrott RA, White P, Watson FG, Wallen R (2007) Covariates affecting spatial variability in bison travel behavior in Yellowstone National Park. Ecological Applications 17: 1411-1423.

3. Zeigenfuss LC, Singer FJ (2003) Ecology of native ungulates in the Jackson valley: habitat selection, interactions with domestic livestock, and effects of herbivory on grassland and willow communities. Fort Collins, Colorado: U.S. Geological Survey, Biological Resources Discipline, Fort Collins Science Center, 2150 Centre Avenue, Building C, Fort Collins, Colorado 80526 and Natural Resources Ecology Laboratory, Colorado State University, Fort Collins, Colorado, 80523. 118 + appendices p.

4. Fischer LS, Gates CC (2005) Comptetition potential between sympatric woodland caribou and wood bison in southwestern Yukon, Canada. Canadian Journal of Zoology 83: 1162 - 1173.

5. Van Vuren DJ (2001) Spatial relations of American bison (*Bison bison*) and domestic cattle in a montane environment. Animal Biodiversity and Conservation 24: 117 - 123.

6. Dancose K, Fortin D, Guo XL (2011) Mechanisms of functional connectivity: the case of free-ranging bison in a forest landscape. Ecological Applications 21: 1871-1885.

7. Duchesne T, Fortin D, Courbin N (2010) Mixed conditional logistic regression for habitat selection studies. Journal of Animal Ecology 79: 548-555.

8. Leverkus SER (2012) Seasonal range use by wood bison in British Columbia. Prepared for: Biritish Columbia Ministry of Natural Resource Operations.

9. Keller BJ (2011) Factors affecting spatial and temporal dynamics of an ungulate assemblage in the Black Hills, South Dakota: University of Missouri--Columbia.

10. Larter NC (1988) Diet and habitat selection of an erupting wood bison population. Vancouver: University of British Columbia.

11. Ranglack DH, du Toit J (2015) Habitat Selection by Free-Ranging Bison in a Mixed Grazing System on Public Land. Rangeland Ecology & Management 68: 349-353.
