# Supplementary material for: Assessing Potential Habitat and Carrying Capacity for Reintroduction of Plains Bison (Bison bison bison) in Banff National Park
Source: PLoS One. 2016 Feb 24;11(2):e0150065. doi: 10.1371/journal.pone.0150065 (PMC4765961; doi:10.1371/journal.pone.0150065)
Supplement: S2 Table — Scores based on standardized rankings for homologous landcover types from previous published studies on bison winter habitat use. Dashes indicate no homologous landcover type was studied. (DOCX) [file pone.0150065.s002.docx]

**S2 Table. Bison (*Bison bison bison*) winter landcover suitability (from 0, low to 1, high) for Banff National Park.** Scores based on standardized rankings for homologous landcover types from previous published studies on bison winter habitat use. Dashes indicate no homologous landcover type was studied. Note also that Ranglack et al. [1] did not present coefficients that could be standardized.

| **Banff Landcover Covariates** | **Bruggeman** | **Zeigenfuss & Francis** | **Fischer & Gates** | **Keller** | **Mean HSI** | **Rank** | **Notes** |
| --- | --- | --- | --- | --- | --- | --- | --- |
| snow/ice | --- | --- | --- | --- | 0.000 | 13 | Assumed to be 0 |
| rock | --- | --- | 0.250 | --- | 0.050 | 12 | Assumed 0.05 |
| open conifer | 0.286 | 0.500 | 0.375 | 0.429 | 0.397 | 5 |  |
| moderate conifer | 0.143 | 0.250 | 0.000 | 0.286 | 0.170 | 7 | Same c. conifer |
| closed conifer | 0.143 | 0.250 | 0.000 | 0.286 | 0.170 | 7 |  |
| mixed forest | 0.429 | 0.375 | 0.500 | 0.143 | 0.362 | 6 |  |
| deciduous | 0.571 | 0.625 | 0.625 | 0.571 | 0.598 | 3 |  |
| herbaceous | 0.857 | 0.875 | 0.875 | 0.857 | 0.866 | 2 |  |
| alpine herbaceous | --- | --- | 0.125 | --- | 0.125 | 9 |  |
| shrub | 0.714 | 0.000 | 0.750 | 0.714 | 0.545 | 4 |  |
| alpine shrub | --- | --- | 0.125 | --- | 0.125 | 9 |  |
| burn forest | 0.000 | --- | --- | 0.000 | 0.000 | 13 |  |
| burn grassland | --- | 0.875 | --- | --- | 0.875 | 1 |  |
| burn shrubland | --- | 0.125 | --- | --- | 0.125 | 9 |  |

References

1. Ranglack DH, du Toit J (2015) Habitat Selection by Free-Ranging Bison in a Mixed Grazing System on Public Land. Rangeland Ecology & Management 68: 349-353.
