# Supplementary material for: Assessing Potential Habitat and Carrying Capacity for Reintroduction of Plains Bison (Bison bison bison) in Banff National Park
Source: PLoS One. 2016 Feb 24;11(2):e0150065. doi: 10.1371/journal.pone.0150065 (PMC4765961; doi:10.1371/journal.pone.0150065)
Supplement: S3 Table — Scores based on standardized rankings for homologous landcover types from previous published studies on bison summer habitat use. Dashes indicate no homologous landcover type was studied. (DOCX) [file pone.0150065.s003.docx]

**S3 Table. Bison (*Bison bison bison*) summer landcover suitability (from 0, low to 1, high) for Banff National Park.** Scores based on standardized rankings for homologous landcover types from previous published studies on bison summer habitat use. Dashes indicate no homologous landcover type was studied.

| **Banff Landcover Covariates** | **Keller** | | **Campbell & Hinkes** | | **Larter** | | **Mean HSI** | | **Rank** | | **Notes** | |
| --- | --- | --- | --- | --- | --- | --- | --- | --- | --- | --- | --- | --- |
| snow/ice | --- | --- | | --- | | 0.000 | | 13 | | Assumed to be 0 | |  |
| rock | --- | --- | | --- | | 0.010 | | 12 | | Assumed nominal | |  |
| open conifer | 0.357 | --- | | --- | | 0.357 | | 6 | |  | |  |
| closed conifer | 0.286 | 0.667 | | 0.000 | | 0.317 | | 7 | |  | |  |
| mixed forest | 0.429 | --- | | 0.200 | | 0.314 | | 8 | |  | |  |
| deciduous | 0.143 | --- | | 0.400 | | 0.271 | | 10 | |  | |  |
| herbaceous | 0.857 | 0.333 | | 0.800 | | 0.663 | | 1 | |  | |  |
| alpine herbaceous | --- | --- | | --- | | 0.300 | | 9 | | Assumed 0.3 | |  |
| shrub | 0.714 | 0.000 | | 0.600 | | 0.438 | | 3 | |  | |  |
| alpine shrub | --- | --- | | --- | | 0.250 | | 11 | | Assumed 0.25 | |  |
| burn forest | 0.000 | 0.833 | | --- | | 0.417 | | 4 | |  | |  |
| burn grassland | --- | 0.500 | | --- | | 0.500 | | 2 | |  | |  |
| burn shrubland | --- | 0.167 | | --- | | 0.400 | | 5 | | Adjusted to 0.4 | |  |

**Additional details:** We assigned snow/ice an HSI value of 0 under the assumption that bison would be extremely unlikely to use permanent snowfields and glaciers in either season. For alpine herbaceous and alpine shrub during summer, while no published studies have examined bison habitat selection or use of these high elevation landcover types, previous archaeological studies report bison skulls found at high elevation alpine meadows in Rocky Mountain National Park [1], in the GYE [2]. Moreover, in the Henry Mountains of Utah, while no homologous alpine landcover types can be found, bison use high elevation mountain ridges during summer [3]. Therefore, we assumed HSI values of alpine herbaceous and alpine shrublands would be half of their low elevation analogues. No previous studies report quantitative data on bison use of burned shrublands during summer. However, during winter, bison showed strong selection for burned shrublands, and bison in Alaska and NWT made extensive use of shrublands during summer. Based on previous studies showing that burned shrublands had high forage value for ungulates [4], we ranked burned shrublands during summer just slightly lower than burned grasslands, but higher than burned forests.

References

1. Fryxell FM. The former range of the Bison in the Rocky Mountains. J Mammal. 1928; 9: 129 – 139.

2. Cannon KP. "They went as high as they choose:" What an isolated skull can tell us about the Biogeography of high-altitude bison. Arct Antarct Alp Res. 2007; 39: 44 – 56.

3. Van Vuren DJ (2001) Spatial relations of American bison (*Bison bison*) and domestic cattle in a montane environment. Animal Biodiversity and Conservation 24: 117 - 123.

4. Sachro LL, Strong WL, Gates CC. Prescribed burning effects on summer elk forage availability in the subalpine zone, Banff National Park. Environ Manage. 2005; 77: 183–193.
