# Supplementary material for: Assessing Potential Habitat and Carrying Capacity for Reintroduction of Plains Bison (Bison bison bison) in Banff National Park
Source: PLoS One. 2016 Feb 24;11(2):e0150065. doi: 10.1371/journal.pone.0150065 (PMC4765961; doi:10.1371/journal.pone.0150065)
Supplement: S4 Table — See Hebblewhite et al. [55] for more details. (DOCX) [file pone.0150065.s004.docx]

**S4 Table.** **Forage biomass (kg/ha) as a function of landcover type, and proportion of landcover type, in the primary and secondary reintroduction areas of Banff National Park, separated into forb, graminoid and shrub biomass.** See [1] for more details.

| **Landcover type** | **Reintro. Area** | |  | | **Biomass (kg/ha)** | | | | |  |
| --- | --- | --- | --- | --- | --- | --- | --- | --- | --- | --- |
|  | **1°** | **2°** | |  | | **Forb** | **Grass** | **Shrub** | **Total** | |
| Snow/ Rock | 0.456 | 0.456 | |  | | 0 | 10 | 10 | 20 | |
| Alpine-Herbaceous | 0.059 | 0.059 | |  | | 1.4 | 73.8 | 188.4 | 263.6 | |
| Alpine-Shrubs | 0.025 | 0.025 | |  | | 1.9 | 154.5 | 370.4 | 526.8 | |
| Burn-Forest | 0.068 | 0.068 | |  | | 3.2 | 359.6 | 506.3 | 869.2 | |
| Burn-Grassland | 0.015 | 0.015 | |  | | 3.6 | 426.2 | 390.6 | 820.4 | |
| Burn-Shrub | 0.002 | 0.002 | |  | | 3.1 | 501.5 | 883.0 | 1387.6 | |
| Closed Conifer | 0.097 | 0.097 | |  | | 0.7 | 37.5 | 979.7 | 1017.9 | |
| Deciduous | 0.001 | 0.001 | |  | | 3.6 | 562.5 | 205.9 | 772.1 | |
| Forest regeneration* | 0 | 0 | |  | | 1.7 | 460.3 | 648.1 | 1110.1 | |
| Herbaceous | 0.023 | 0.023 | |  | | 2.3 | 364.6 | 518.9 | 885.8 | |
| Mixed forest | 0.004 | 0.004 | |  | | 1.8 | 143.8 | 1368.1 | 1513.7 | |
| Moderate Conifer | 0.167 | 0.167 | |  | | 1.2 | 84.2 | 1109.8 | 1195.3 | |
| Open Conifer | 0.048 | 0.048 | |  | | 1.6 | 179.2 | 756.1 | 936.9 | |
| Shrubs | 0.035 | 0.035 | |  | | 2.4 | 365.4 | 1085.0 | 1452.7 | |
| **Total** | 1 | 1 | |  | | 2.3 | 271.9 | 757.0 | 1031.2 | |

*No cutblocks occur within the areas evaluated for bison reintroduction

References

1. Hebblewhite M, Merrill E, McDermid G. A multi-scale test of the forage maturation hypothesis in a partially migratory ungulate population. Ecol Monogr. 2008; 78: 141–166.
