# Supplementary material for: Assessing Potential Habitat and Carrying Capacity for Reintroduction of Plains Bison (Bison bison bison) in Banff National Park
Source: PLoS One. 2016 Feb 24;11(2):e0150065. doi: 10.1371/journal.pone.0150065 (PMC4765961; doi:10.1371/journal.pone.0150065)
Supplement: S5 Table — (DOCX) [file pone.0150065.s005.docx]

**S5 Table.** **Summary of literature values for bison (*Bison bison*) intake rate estimation including reported age and sex structure that were used for bison carry capacity estimation for Banff National Park.**

| **Study** | **Study area** | **Intake rate estimation** |
| --- | --- | --- |
| Kuzyk et al. [1] | Elk Island National Park | Reported 10.4 kg/day for bison based on a weighted population distribution was assumed to be 50:50 with an average mass adjusted for age-distribution of 615kg, for 1.7% adjusted intake rate as a function of body mass/day |
| Strong and Gates [2] | Wood Buffalo National Park | Reported that >13.3 kg of forage was required to maintain an average wood bison with an average body mass of 533kg at a year-round daily consumption rate of 2.5% of body weight. Forage requirements during winter were increased to 4% of body mass to account for the effects of trampling and snow hardening (1.5%) following disturbance by foraging activity |
| Hamilton [3] | Wood Buffalo National Park | Reported intake rates of 10.4 kg/day for wood bison, and sex ratio’s of 0.36 bulls, 0.36 cows, 0.14 yearlings and 0.14 calves weighing 907, 435, 272 and 158 kg, respectively. |
| Turner et al. [4] | Yellowstone National Park | Reported that the sex ratio of the population was 0.18 bison calves, 0.38 bison cows and 0.42 bison bulls, and that bison body mass at the beginning of winter was 159kg for calves, 409 for cows and 909 kg for bison bulls. Using an assumed maximum daily intake rate of 2.5% for adults and 3% for calves, and body mass reported above, daily intake rates were 4.78 kg/day, 10.15 kg/day and 22.73 kg/day for calves, cows and bulls, respectively. |
| Coughenour [5] | Yellowstone National Park | Reported that mean body weights during winter were assumed to be 160kg for calves, 300 kg for yearlings, 450 kg for adult females and 650 kg for bulls, based on an complex age/sex distribution given in Table 8. Intake rates averaged 2.5%. The only study that adjusted for overwinter mass loss of 18%, but here we did not adjust for declining body mass over winter because of the uncertainty in starting body mass values themselves. |
| Keller [6] | Custer State Park (the Black Hills), SD | Reported winter sex ratios of 12% adult male, 53% adult female, 6% yearling male, 8% yearling female, 9 juvenile male and 13% juvenile females. Body mass, respectively, is reported as 800kg, 470kg, 300kg, 263 kg, 295kg, and 261 kg. Based on these body mass values, the % daily intake rates Keller used to develop her carrying capacity model were 1.7% for adult males and yearling males and females, 2.7% for adult females (including the costs of reproduction) and 3.1% for juveniles. |

References

1. Kuzyk GW, Nool NL, Bork EW, Bampfylde C, Franke A, Hudson RJ. Estimating economic carrying capacity for an ungulate guild in western Canada. The Open Conservation Biology Journal. 2009; 3: 24–35.

2. Strong WL, Gates CC. Wood bison population recovery and forage availability in northwestern Canada. J Environ Manage. 2009; 90: 434–440.

3. Hamilton SG. Estimating winter carrying capacity for bison in Wood Buffalo National Park. Edmonton: University of Alberta. 2005.

4. Turner MG, Wu Y, Wallace LL, Romme WH, Brenkert A. Simulating winter interactions among ungulates, vegetation, and fire in northern Yellowstone Park. Ecol Appl. 1994; 4: 472–496.

5. Coughenour MB. Bison and Elk in Yellowstone National Park - Linking Ecosystem, Animal Nutrition, and Population Processes. Natural Resource Ecology Laboratory, Colorado State University, Fort Collins, Colorado. 2005; 38 + appendices p.

6. Keller BJ. Factors affecting spatial and temporal dynamics of an ungulate assemblage in the Black Hills, South Dakota: University of Missouri--Columbia. 2011.
